# (5-Bromo-2-hydroxy­phen­yl)(phen­yl)methanone

**DOI:** 10.1107/S160053680803969X

**Published:** 2008-11-29

**Authors:** Chang-Zheng Zheng, Chang-You Ji, Xiu-Li Chang, Li-Qin Zhang

**Affiliations:** a College of Environmental and Chemical Engineering, Xi’an Polytechnic University, 710048 Xi’an, Shaanxi, People’s Republic of China; b Department of Materials Science and Chemical Engineering, Sichuan University of Science and Engineering , 643000 Zigong, Sichuan, People’s Republic of China

## Abstract

In the title compound, C_13_H_9_BrO_2_, the dihedral angle between the aromatic ring planes is 53.6 (1)°. The crystal structure is stabilized by intra­molecular O—H⋯O and inter­molecular C—H⋯O hydrogen bonding and C—H⋯π inter­actions.

## Related literature

For the ability of aroylhydrazones to coordinate to metal ions and their biological activity, see: Singh *et al.* (1982 ▶); Salem (1998 ▶); Carcelli *et al.* (1995 ▶).
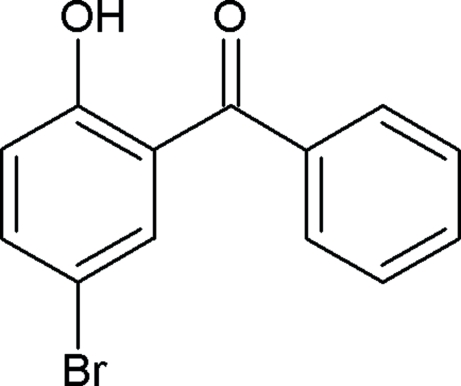


## Experimental

### 

#### Crystal data

C_13_H_9_BrO_2_
*M* *_r_* = 277.11
Monoclinic, 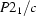

*a* = 15.9510 (18) Å
*b* = 5.8956 (6) Å
*c* = 12.1260 (14) Å
β = 106.166 (2)°
*V* = 1095.2 (2) Å^3^
*Z* = 4
Mo *K*α radiation
μ = 3.73 mm^−1^
*T* = 298 K
0.15 × 0.10 × 0.06 mm

#### Data collection

Siemens SMART CCD area-detector diffractometer
Absorption correction: multi-scan (*SADABS*; Sheldrick, 1996 ▶) *T*_min_ = 0.604, *T*_max_ = 0.807
5479 measured reflections
1933 independent reflections
1578 reflections with *I* > 2σ(*I*)
*R*_int_ = 0.027

#### Refinement

*R*[*F* ^2^ > 2σ(*F* ^2^)] = 0.029
*wR*(*F* ^2^) = 0.072
*S* = 1.03
1933 reflections
146 parameters
H-atom parameters constrained
Δρ_max_ = 0.33 e Å^−3^
Δρ_min_ = −0.52 e Å^−3^

### 

Data collection: *SMART* (Siemens, 1996 ▶); cell refinement: *SAINT* (Siemens, 1996 ▶); data reduction: *SAINT* (Siemens, 1996 ▶); program(s) used to solve structure: *SHELXS97* (Sheldrick, 2008 ▶); program(s) used to refine structure: *SHELXL97* (Sheldrick, 2008 ▶); molecular graphics: *SHELXTL* (Sheldrick, 2008 ▶); software used to prepare material for publication: *SHELXTL* (Sheldrick, 2008 ▶).

## Supplementary Material

Crystal structure: contains datablocks I, global. DOI: 10.1107/S160053680803969X/at2684sup1.cif

Structure factors: contains datablocks I. DOI: 10.1107/S160053680803969X/at2684Isup2.hkl

Additional supplementary materials:  crystallographic information; 3D view; checkCIF report

## Figures and Tables

**Table 1:** Hydrogen-bond geometry (Å, °)

*D*—H⋯*A*	*D*—H	H⋯*A*	*D*⋯*A*	*D*—H⋯*A*
O1—H1⋯O2	0.82	1.85	2.569 (3)	146
C3—H3⋯O1^i^	0.93	2.60	3.488 (3)	160
C12—H12⋯*Cg*1^ii^	0.93	2.93	3.596 (3)	130

Symmetry codes: (i) 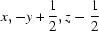; (ii) 
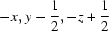
. *Cg*1 is the centroid of the C8–C13 ring.

**Fig. 1.**
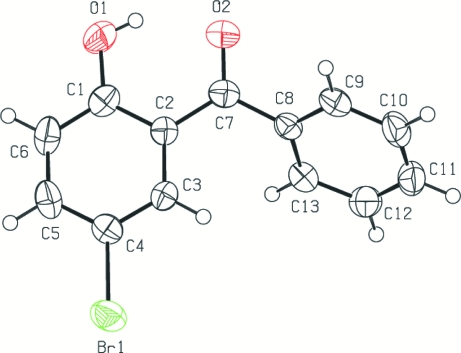
The molecular structure of compound (I), showing the atom-numbering scheme. Displacement ellipsoids are drawn at the 50% probability level.

**Fig. 2.**
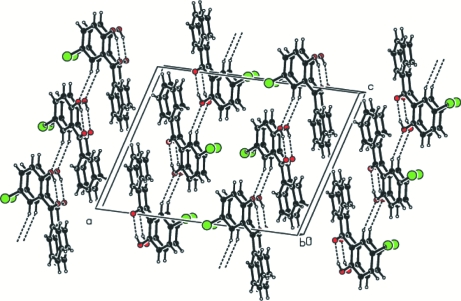
The crystal packing of (I), viewed down the b axis. Dashed lines show intra-and intermolecular hydrogen bonds.

## supplementary crystallographic information

### Comment

The chemistry of aroylhydrazones continues to attract much attention due to their coordination ability to metal ions (Singh *et al.*, 1982; Salem, 1998) and their biological activity (Singh *et al.*, 1982; Carcelli *et al.*, 1995). As an extension of work on the structural characterization of aroylhydrazone derivatives, the title compound, (I), was synthesized and its crystal structure is reported here.

The title molecule displays a *trans* conformation with respect to the C7=O2 double bond (Fig. 1). The crystal structure is stabilized by intramolecular O—H···O and intermolecular C—H···O hydrogen bonding and C-H···π interactions (Table 1. and Fig. 2).

### Experimental

The benzoyl chloride (0.01 mol, 1.4057 g) and the 4-bromophenol, heated up in the oil bath, the reaction mixture was refluxed for 6 h with stirring. Then anhydrous aluminium trichloride (3 mol, 1:3) was added, the backflow agitation responds for 4 h (yield 80%). The compound (2.0 mmol, 0.67 g) was dissolved in dimethylformamide (30 ml) and kept at room temperature for 30 d to obtain brown single crystals suitable for X-ray diffraction.

### Refinement

All H atoms were positioned geometrically and treated as riding on their parent atoms,with C—H(aromatic) = 0.93 Å, O—H = 0.82 Å, and with *U*_iso_(H) = 1.2U_eq_(C) and 1.5U_eq_(O).

### Crystal data

**Table:**

C_13_H_9_BrO_2_	*F*_000_ = 552
*M**_r_* = 277.11	*D*_x_ = 1.681 Mg m^−^^3^
Monoclinic, *P*2_1_/*c*	Mo *K*α radiation λ = 0.71073 Å
Hall symbol: -P 2ybc	Cell parameters from 2043 reflections
*a* = 15.9510 (18) Å	θ = 2.7–23.4º
*b* = 5.8956 (6) Å	µ = 3.73 mm^−^^1^
*c* = 12.1260 (14) Å	*T* = 298 K
β = 106.166 (2)º	Block, yellow
*V* = 1095.2 (2) Å^3^	0.15 × 0.10 × 0.06 mm
*Z* = 4	

### Data collection

**Table:**

Siemens SMART CCD area-detector diffractometer	1933 independent reflections
Radiation source: fine-focus sealed tube	1578 reflections with *I* > 2σ(*I*)
Monochromator: graphite	*R*_int_ = 0.027
*T* = 273(2) K	θ_max_ = 25.1º
φ and ω scans	θ_min_ = 2.7º
Absorption correction: multi-scan(SADABS; Sheldrick, 1996)	*h* = −18→19
*T*_min_ = 0.604, *T*_max_ = 0.807	*k* = −4→7
5479 measured reflections	*l* = −14→14

### Refinement

**Table:**

Refinement on *F*^2^	Secondary atom site location: difference Fourier map
Least-squares matrix: full	Hydrogen site location: inferred from neighbouring sites
*R*[*F*^2^ > 2σ(*F*^2^)] = 0.029	H-atom parameters constrained
*wR*(*F*^2^) = 0.073	*w* = 1/[σ^2^(*F*_o_^2^) + (0.0296*P*)^2^ + 0.7566*P*] where *P* = (*F*_o_^2^ + 2*F*_c_^2^)/3
*S* = 1.03	(Δ/σ)_max_ < 0.001
1933 reflections	Δρ_max_ = 0.33 e Å^−^^3^
146 parameters	Δρ_min_ = −0.52 e Å^−^^3^
Primary atom site location: structure-invariant direct methods	Extinction correction: none

### Special details

**Table:**

Geometry. All e.s.d.'s (except the e.s.d. in the dihedral angle between two l.s. planes) are estimated using the full covariance matrix. The cell e.s.d.'s are taken into account individually in the estimation of e.s.d.'s in distances, angles and torsion angles; correlations between e.s.d.'s in cell parameters are only used when they are defined by crystal symmetry. An approximate (isotropic) treatment of cell e.s.d.'s is used for estimating e.s.d.'s involving l.s. planes.
Refinement. Refinement of *F*^2^ against ALL reflections. The weighted *R*-factor *wR* and goodness of fit *S* are based on *F*^2^, conventional *R*-factors *R* are based on *F*, with *F* set to zero for negative *F*^2^. The threshold expression of *F*^2^ > σ(*F*^2^) is used only for calculating *R*-factors(gt) *etc*. and is not relevant to the choice of reflections for refinement. *R*-factors based on *F*^2^ are statistically about twice as large as those based on *F*, and *R*- factors based on ALL data will be even larger.

### Fractional atomic coordinates and isotropic or equivalent isotropic displacement parameters (Å^2^)

**Table:**

	*x*	*y*	*z*	*U*_iso_*/*U*_eq_	
Br1	0.42247 (2)	−0.27591 (5)	0.49702 (3)	0.05526 (14)	
O1	0.28452 (15)	0.5186 (4)	0.70204 (17)	0.0548 (6)	
H1	0.2528	0.5974	0.6517	0.082*	
O2	0.18991 (14)	0.6145 (4)	0.49878 (17)	0.0537 (6)	
C1	0.31010 (19)	0.3343 (5)	0.6540 (2)	0.0404 (7)	
C2	0.27504 (17)	0.2828 (4)	0.5366 (2)	0.0330 (6)	
C3	0.30905 (17)	0.0971 (4)	0.4914 (2)	0.0336 (6)	
H3	0.2877	0.0616	0.4139	0.040*	
C4	0.37381 (18)	−0.0324 (5)	0.5610 (2)	0.0384 (6)	
C5	0.40544 (19)	0.0138 (6)	0.6778 (3)	0.0493 (8)	
H5	0.4478	−0.0789	0.7248	0.059*	
C6	0.37369 (19)	0.1969 (6)	0.7229 (2)	0.0485 (8)	
H6	0.3952	0.2292	0.8008	0.058*	
C7	0.20810 (18)	0.4294 (5)	0.4636 (2)	0.0362 (6)	
C8	0.16079 (17)	0.3613 (5)	0.3442 (2)	0.0340 (6)	
C9	0.15422 (19)	0.5167 (5)	0.2563 (2)	0.0415 (7)	
H9	0.1816	0.6569	0.2718	0.050*	
C10	0.1068 (2)	0.4626 (6)	0.1452 (3)	0.0503 (8)	
H10	0.1042	0.5646	0.0858	0.060*	
C11	0.0636 (2)	0.2594 (6)	0.1225 (3)	0.0510 (8)	
H11	0.0310	0.2250	0.0481	0.061*	
C12	0.06851 (19)	0.1062 (5)	0.2098 (3)	0.0466 (7)	
H12	0.0381	−0.0300	0.1942	0.056*	
C13	0.11812 (18)	0.1530 (5)	0.3204 (2)	0.0385 (6)	
H13	0.1230	0.0464	0.3784	0.046*	

### Atomic displacement parameters (Å^2^)

**Table:**

	*U*^11^	*U*^22^	*U*^33^	*U*^12^	*U*^13^	*U*^23^
Br1	0.0478 (2)	0.0490 (2)	0.0675 (2)	0.01286 (15)	0.01368 (16)	−0.00212 (16)
O1	0.0628 (15)	0.0608 (15)	0.0403 (11)	0.0066 (11)	0.0136 (11)	−0.0127 (10)
O2	0.0641 (15)	0.0420 (13)	0.0519 (13)	0.0133 (10)	0.0108 (11)	−0.0070 (10)
C1	0.0385 (16)	0.0481 (17)	0.0381 (15)	−0.0056 (13)	0.0162 (13)	−0.0043 (13)
C2	0.0326 (14)	0.0341 (15)	0.0329 (13)	−0.0019 (11)	0.0102 (11)	0.0019 (11)
C3	0.0336 (15)	0.0363 (15)	0.0309 (14)	−0.0059 (12)	0.0091 (12)	−0.0005 (12)
C4	0.0330 (15)	0.0396 (16)	0.0450 (16)	0.0014 (12)	0.0146 (13)	0.0027 (13)
C5	0.0364 (17)	0.065 (2)	0.0425 (17)	0.0083 (15)	0.0039 (13)	0.0110 (15)
C6	0.0390 (17)	0.073 (2)	0.0306 (15)	0.0016 (16)	0.0054 (13)	0.0008 (15)
C7	0.0400 (16)	0.0324 (15)	0.0399 (15)	−0.0013 (12)	0.0172 (13)	0.0024 (12)
C8	0.0312 (14)	0.0339 (15)	0.0377 (15)	0.0057 (12)	0.0109 (12)	0.0017 (12)
C9	0.0457 (18)	0.0331 (16)	0.0476 (17)	0.0047 (13)	0.0160 (14)	0.0075 (13)
C10	0.055 (2)	0.057 (2)	0.0378 (17)	0.0142 (16)	0.0116 (15)	0.0143 (15)
C11	0.0438 (17)	0.064 (2)	0.0393 (16)	0.0131 (16)	0.0013 (13)	−0.0034 (16)
C12	0.0351 (16)	0.0453 (18)	0.0565 (19)	−0.0009 (13)	0.0079 (14)	−0.0071 (15)
C13	0.0360 (15)	0.0381 (16)	0.0421 (16)	0.0035 (12)	0.0117 (13)	0.0056 (13)

### Geometric parameters (Å, °)

**Table:**

Br1—C4	1.898 (3)	C6—H6	0.9300
O1—C1	1.348 (3)	C7—C8	1.489 (4)
O1—H1	0.8200	C8—C9	1.388 (4)
O2—C7	1.235 (3)	C8—C13	1.395 (4)
C1—C6	1.382 (4)	C9—C10	1.386 (4)
C1—C2	1.411 (4)	C9—H9	0.9300
C2—C3	1.400 (4)	C10—C11	1.371 (4)
C2—C7	1.464 (4)	C10—H10	0.9300
C3—C4	1.370 (4)	C11—C12	1.377 (4)
C3—H3	0.9300	C11—H11	0.9300
C4—C5	1.391 (4)	C12—C13	1.382 (4)
C5—C6	1.370 (4)	C12—H12	0.9300
C5—H5	0.9300	C13—H13	0.9300
			
C1—O1—H1	109.5	O2—C7—C8	118.0 (2)
O1—C1—C6	118.1 (3)	C2—C7—C8	121.0 (2)
O1—C1—C2	121.8 (3)	C9—C8—C13	119.6 (3)
C6—C1—C2	120.1 (3)	C9—C8—C7	118.6 (2)
C3—C2—C1	118.4 (2)	C13—C8—C7	121.6 (2)
C3—C2—C7	121.4 (2)	C10—C9—C8	120.0 (3)
C1—C2—C7	120.0 (2)	C10—C9—H9	120.0
C4—C3—C2	120.1 (2)	C8—C9—H9	120.0
C4—C3—H3	119.9	C11—C10—C9	120.3 (3)
C2—C3—H3	119.9	C11—C10—H10	119.9
C3—C4—C5	121.1 (3)	C9—C10—H10	119.9
C3—C4—Br1	119.5 (2)	C10—C11—C12	120.1 (3)
C5—C4—Br1	119.4 (2)	C10—C11—H11	120.0
C6—C5—C4	119.4 (3)	C12—C11—H11	120.0
C6—C5—H5	120.3	C11—C12—C13	120.6 (3)
C4—C5—H5	120.3	C11—C12—H12	119.7
C5—C6—C1	120.8 (3)	C13—C12—H12	119.7
C5—C6—H6	119.6	C12—C13—C8	119.4 (3)
C1—C6—H6	119.6	C12—C13—H13	120.3
O2—C7—C2	121.0 (2)	C8—C13—H13	120.3

### Hydrogen-bond geometry (Å, °)

**Table:**

*D*—H···*A*	*D*—H	H···*A*	*D*···*A*	*D*—H···*A*
O1—H1···O2	0.82	1.85	2.569 (3)	146
C3—H3···O1^i^	0.93	2.60	3.488 (3)	160
C12—H12···Cg1^ii^	0.93	2.93	3.596 (3)	130

Symmetry codes: (i) *x*, −*y*+1/2, *z*−1/2; (ii) −*x*, *y*−1/2, −*z*+1/2.
